# Sex-dependent associations of childhood neglect and bodyweight across the life span

**DOI:** 10.1038/s41598-019-41367-y

**Published:** 2019-03-25

**Authors:** M. Ernst, A. N. Tibubos, A. Werner, M. E. Beutel, P. L. Plener, J. M. Fegert, E. Brähler

**Affiliations:** 1 Department of Psychosomatic Medicine and Psychotherapy, University Medical Center, Johannes Gutenberg-University Mainz, Untere Zahlbacher Str. 8, 55131 Mainz, Germany; 2 Department of Child and Adolescent Psychiatry/Psychotherapy, University of Ulm, Steinhoevelstr. 5, 89075 Ulm, Germany; 3 Department of Child and Adolescent Psychiatry, Medical University of Vienna, Waehringerguertel 18–20, 1090 Vienna, Austria

## Abstract

Eating disorders and weight problems across the life span have been linked to adverse childhood experiences. Previous research often focused on child abuse and omitted investigating effects of child neglect. The present study evaluates effects of neglect on bodyweight across the life span and how emotional neglect and bodyweight are linked via mental distress. Within a large survey representative of the German population (*N* = 2,500), individuals completed measures of mental distress, childhood trauma, and height and weight. We conducted logistic regression analyses on bodyweight extremes and a moderated mediation analysis. In men, physical neglect aggravated the risk to be underweight. In women, emotional neglect was linked to severe obesity. In both sexes, emotional neglect was related to mental distress. We found an indirect effect of emotional neglect on bodyweight via mental distress, however, it was only present in women. Our results attest to long-term consequences of adverse early experiences. We showed a possible mechanism for women’s higher vulnerability towards eating disorders. In general, investigations of eating and weight disorders should also include men and employ sex-specific methods of analyses. Lastly, neglect should also receive more attention to prevent suffering and negative sequelae over the life span.

## Introduction

Since the first landmark study investigating adverse effects of child maltreatment by Felitti, *et al*.^1^, a growing body of research has focused on the consequences of early, potentially traumatic events^2,3^. Childhood trauma represents a major health issue in the US and around the world e.g.^4^. A recent representative survey of the German population using the established taxonomy of the Childhood Trauma Questionnaire CTQ^5^ which differentiates emotional, physical, and sexual abuse, and physical and emotional neglect, found that almost a third of participants had suffered at least one type of child maltreatment^6^. Maltreatment was associated with lower educational attainment, employment status, and income. This was true for abuse and neglect. Prospective, large scale studies with long follow-up times consistently showed a profound negative impact of maltreatment on development, educational and social attainment, and behavioral problems such as criminal offending^7^, and substantial physical^8^ and mental^9–11^ health risks.

In fact, complex trauma-dependent developmental alterations pertain to the psychological, interpersonal, physiological, and neurological domain. Survivors of maltreatment show aberrations with regard to the stress-response-system, metabolic risk factors such as inflammation^12^ and dyslipidemia^13^, and have an elevated risk to suffer from cardiovascular diseases, diabetes, and autoimmune disorders^14,15^.

Substantial evidence exists for the connection of eating disorders and weight problems with childhood adversities^16–19^. In particular, effects of sexual and physical abuse have drawn attention: Beutel, *et al*.^20^ found a link between sexual abuse and obesity in a large number of female psychosomatic inpatients. This finding corroborates previous research by Wonderlich, *et al*.^21^, Williamson, *et al*.^22^, and Brewerton, *et al*.^23^, the latter citing molestation and physical abuse as risk factors for an early onset of binge eating disorder. A review^24^ confirmed strong links of childhood physical and sexual abuse and obesity in adulthood. Childhood trauma has also been identified as a contributing factor to eating disorders entailing lower weight, such as bulimia and anorexia^25,26^. The connection was confirmed for sexual, physical, and emotional abuse^27^.

The contribution of *neglect* to eating and weight problems is less clear. A recent review by Molendijk, *et al*.^17^ attested to high prevalence rates (21–59%) of any type of childhood trauma in individuals with eating disorders. However, in a sample of 142 young female psychology students, neither emotional nor physical neglect contributed to disordered eating^28^. Beutel, *et al*.^20^ found no connection of emotional neglect and obesity, however, physical neglect aggravated women’s risk for obesity, even after adjusting for depression. Likewise, in a sample of 73 mostly female patients treated for eating disorders, Kong and Bernstein^29^ confirmed an effect of childhood physical neglect on disordered eating.

An investigation of a community-based sample of mothers and their children including Child Protective services data yielded that physical and emotional neglect elevated the offspring’s risk to suffer from eating and/or weight problems^16^. Along the same lines, within the National Comorbidity Survey emotional neglect was related to a lifetime diagnosis of any kind of eating disorder^30^. However, comparisons with other studies are difficult due to the investigation’s qualitative nature and its lack of a psychometrically validated instrument.

Thus, effects of childhood neglect should be addressed in more depth. Although perhaps a more subtle form of child maltreatment than beatings or sexual abuse, it interferes with children’s emotional development in important ways^14,24,31,32^.

Emotion regulation difficulties are a centrepiece of etiological models of eating disorders. Disturbances, e.g. in the aftermath of childhood trauma, render individuals susceptible to a range of mental disorders and distress^33,34^. Trauma-dependent alterations at the brain level relate to structural and functional changes in regions implicated in emotion perception as well as regulation – and they were also present in neglected individuals^35–37^. Correspondingly, eating psychopathology has been shown to be used as a means of regulating negative emotional states, especially by individuals who lack more productive coping strategies^38,39^.

The mediating role of emotion dysregulation (the absence of adaptive coping strategies in the face of negative emotional states as measured by the Difficulties in Emotion Regulation Scale (DERS) by Gratz and Roemer^40^) between childhood abuse and disordered eating has previously been confirmed in a sample of young female students^28^. However, questions remain: it is unclear whether the model can be extended onto large population-based samples and how an individual’s sex shapes the outcomes of childhood trauma. Especially regarding eating and weight problems, previous investigations often focused on women.

The present study aims at expanding previous research on long-term effects of childhood emotional and physical neglect by investigating a representative sample from the community, using sex-specific analyses, considering extreme bodyweight categories on both ends of the spectrum (i.e. underweight and severely obese individuals). First, we investigated weight extremes in men and women using logistic regression analyses. Focusing on emotional neglect, in the second part of the paper, the proposed mediating role of mental distress is tested empirically. Building on previous research, we hypothesized that mental distress links childhood adversity and bodyweight extremes, as the latter can be brought about by distressed individuals using eating or fasting as inadequate coping behaviors. Thus, we aimed to expand previous research by empirically testing whether mental distress symptoms are indeed the proposed link between early interpersonal difficulties and eating and weight problems later in life.

The following questions shall be answered:

How does childhood (emotional and physical) neglect affect bodyweight in both sexes across the life span?
a) Does current mental distress mediate the effects of childhood emotional neglect on bodyweight in adulthood?

   b) Is this indirect effect moderated by sex?

**Figure 1:**
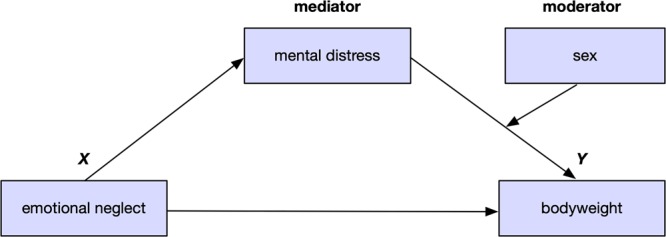
Depiction of the theoretical model. The effect of emotional neglect on bodyweight is mediated by mental distress and moderated by sex.

The statistical model at the basis of questions 2 (a, b) is displayed in Fig. 1.

## Results

### Sample description

In the following, we report survey results of 2,510 individuals. 53.35% of participants were women. Means, standard deviations, and correlations of measures of interest are presented in Table 1. Mean age was 48.4 (SD 18.2) (ranging from 14 to 94) years and mean BMI was 25.76 (SD 4.67) (ranging from 11.47 to 67.76). A higher level of emotional neglect was positively related to increased mental distress, higher age, and higher BMI. BMI was higher for older participants, men, and those with a lower income.

**Table 1:** Means, standard deviations, and correlations among measures.

Variable	Mean (SD)/percentage	Physical neglect	Mental distress	Age	Sex	Income	BMI
Emotional neglect	9.48 (4.43)	0.636**	0.263**	0.082**	0.002	0.013	0.071**
Physical neglect	9.48 (4.44)		0.244**	0.241**	−0.027	0.036	0.061**
Mental distress	1.38 (2.16)			0.028	0.114**	−0.002	0.020
Age	48.4 (18.2)				0.031	0.243**	0.191**
Sex	1_Men_ = 46.65 2_Women_ = 53.35					−0.084**	−0.132**
Income	1.55 (0.55)						−0.016
BMI	25.76 (4.68)						

*Note*. Bivariate analyses are Pearson product-moment correlations, Spearman’s Rho for categorical variables. *p ≤ 0.05, ** *p* ≤ 0.01. Statistics of categorical variables indicate percentages. Emotional neglect and physical neglect: CTQ subscales, range for each: 5–25. Mental distress: PHQ-4 total score, range 0–12. Cut-off according to Löwe, *et al*.^65^: low mental distress: 0–5, high mental distress: 6–12. Income: Equivalised income calculated according to the OECD guideline^67^: household income/√(people in household); household income per month: 1 =< 1,250€, 2 = 1,250–2,500€, 3 => 2,500€. *N* = 2,404.

### Logistic regression analyses on severe obesity and underweight

In order to answer research question 1 concerning the relationship of different types of neglect and weight extremes across the life span, we conducted sex-specific logistic regression analyses (controlling for age and equivalised income). They yielded different predictors for severe obesity and underweight in men and women (see Table 2). In men, neither emotional nor physical neglect was related to severe obesity. Physical neglect was associated with underweight in men.

**Table 2:** Associations of different types of childhood maltreatment and weight extremes in men and women (adjusted for age, equivalised income, and the respective other four types of childhood adversity assessed by the CTQ).

	Severe Obesity			Underweight		
	*OR* (95% CI)	Nagelkerke *R²*	*p*	*OR* (95% CI)	Nagelkerke *R²*	*p*
**Men**						
Emotional neglect	0.00 (0.00–0.00)	0.027	0.99	1.51 (0.07–33.59)	0.233	0.794
Physical neglect	0.88 (0.20–3.79)	0.027	0.86	5.30 (1.10–25.47)	0.233	**0.037**
**Women**						
Emotional neglect	3.30 (1.07–10.22)	0.055	**0.038**	3.22 (0.19–53.87)	0.057	0.416
Physical neglect	1.16 (0.46–2.95)	0.055	0.758	0.45 (0.06–3.63)	0.057	0.457

*Note*. Severe obesity: BMI >= 35. Underweight: BMI < 18.5. *N* = 2404. Emotional neglect: Following the norms by Häuser, *et al*.^45^, total score of the respective CTQ subscale ≥15. Physical neglect: Following the norms by Häuser, *et al*.^45^, total score of the respective CTQ subscale ≥ 10.

In women, emotional neglect statistically predicted severe obesity. The association was positive, i.e. severe obesity was related to having experienced relevant levels of neglect. Physical and emotional neglect failed to predict underweight in women.

### Moderated mediation analysis: Testing the effect of emotional neglect on bodyweight via mental distress

To test our hypotheses that emotional neglect has an indirect effect on bodyweight via mental distress and that this effect further differs dependent on sex, we conducted a moderated mediation model.

**Figure 2:**
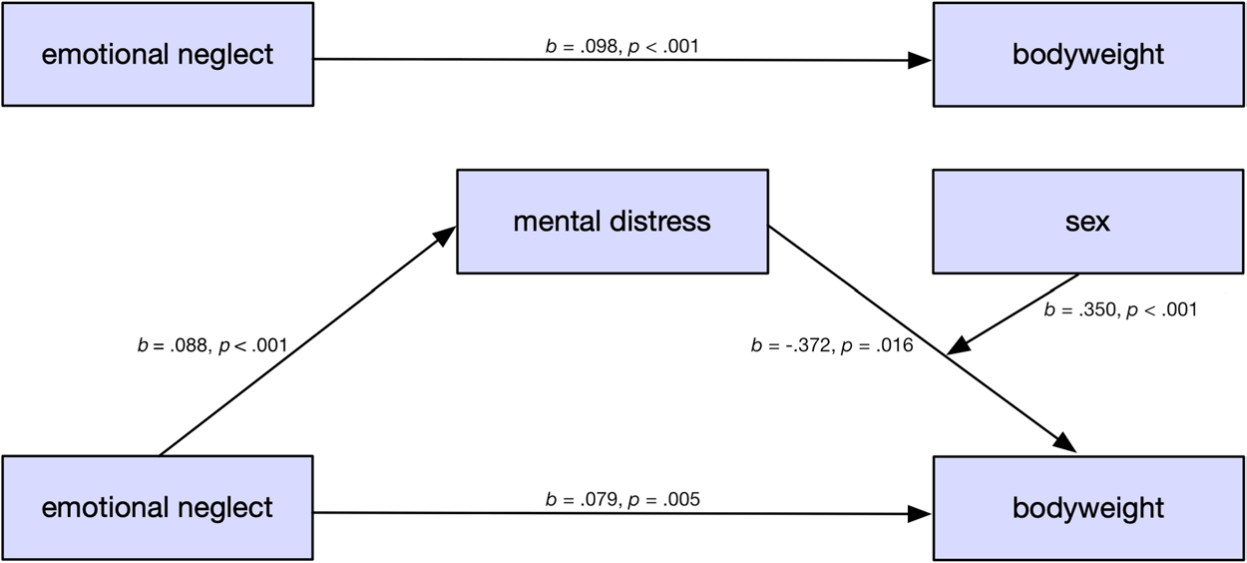
Depiction of the unmediated effect of emotional neglect on bodyweight (model 0, top), and the full moderated mediation model (model 2, bottom). The effect was partially mediated by distress, so that the direct effect (*b* = 0.098, *p* < 0.001) observed in model 0 was smaller in model 2 (*b* = 0.079, *p* = 0.005).

Results are displayed in Table 3 and Fig. 2.

**Table 3:** Results of the moderated mediation analysis on BMI.

	Coeff.	SE	95% CI (L, U)	T	*p*
**Model 0: BMI as criterion**					
Constant	23.291	0.399	22.908/24.473	59.363	<**0.001**
Emotional neglect	0.098	0.028	0.043/0.152	3.527	<**0.001**
***Covariates***					
Age	0.053	0.006	0.042/0.064	9.543	<**0.001**
Income	−0.478	0.176	−0.823/−0.133	−2.720	**0.007**
Other adversities (sum)	−0.100	0.043	−0.185/0.015	−2.308	**0.021**
**Model 1: Mental Distress as criterion**					
Constant	0.354	0.182	−0.003/0.712	1.945	0.052
Emotional Neglect	0.088	0.013	0.063/0.113	6.945	<**0.001**
***Covariates***					
Age	0.001	0.003	−0.004/0.006	0.339	0.902
Income	−0.501	0.175	−0.845/−0.157	−2.858	**0.004**
Other adversities (sum)	−0.115	0.043	−0.199/0.031	−2.677	**0.008**
**Model 2: BMI as criterion**					
Constant	25.749	0.530	24.710/26.788	48.611	<**0.001**
Emotional Neglect	0.079	0.028	0.024/0.134	2.837	**0.005**
Mental Distress	−0.372	0.154	−0.673/−0.070	−2.418	**0.016**
Sex	−1.358	0.221	−1.792/−0.924	−6.133	<**0.001**
***Covariates***					
Age	0.054	0.005	0.044/0.065	9.902	<**0.001**
Income	−0.501	0.175	−0.861/−0.177	−2.978	**0.003**
Other adversities (sum)	0.028	0.030	−0.845/−0.157	−2.858	**0.004**
***Interaction terms***					
Distress × sex	0.350	0.091	0.172/0.528	3.854	<**0.001**

*Note*. EN: Emotional neglect according to the CTQ (range 5–25). Income: Equivalised income calculated according to the OECD guideline^67^: household income/√(people in household); household income per month: 1 = < 1,250€, 2 = 1,250–2500€, 3 = > 2,500€. *N* = 2,404.

**Figure 3:**
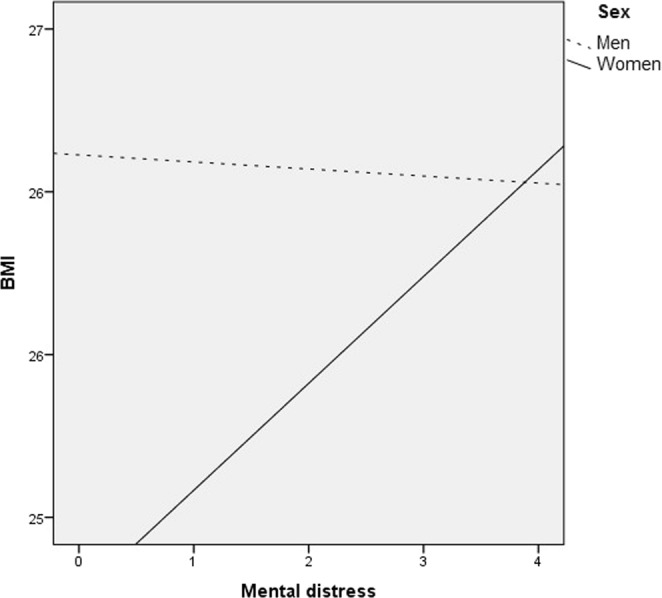
Association of mental distress and BMI as a function of sex: The drawn through line represents a significant positive association between distress and BMI among women (*b* = 0.328 [95% CI 0.215–0.441], *p* < 0.001). The dotted line indicates a nonsignificant association of BMI and distress among men (*b* = −0.022 [95% CI −0.163–0.120], *p* = 0.764).

First, we tested a regression model (model 0) predicting BMI which included only emotional neglect (*p* < 0.001), and the covariates age (*p* < 0.001), equivalised income (*p* = 0.007), and sum of other childhood adversities (*p* = 0.021) (*R*^2^ = 0.042, *F* (4, 2289) = 25.991, *p* < 0.001). Note that mediator (mental distress) and moderator (sex) were not included. The model yielded a significant positive effect of emotional neglect on BMI. It is visualized in the upper half of Fig. 3. Thus, it established a statistically relevant connection.

We then tested whether the mediator (mental distress) was, in accordance with our hypotheses, significantly predicted by emotional neglect (and covariates age, equivalised income, and sum of other childhood adversities). This was the case (model 1: *R*^2^ = 0.0724, *F*(4, 2278) = 44.477, *p* < 0.001).

The full model (model 2) included the covariates age, equivalised income, and sum of other childhood adversities, the proposed mediator mental distress, and the proposed moderator sex (i.e. the interaction term mental distress x sex). It predicted BMI (*R*^2^ = 0.0652, *F*(7, 2275) = 22.655, *p* < 0.001) with the significant predictors age, equivalised income, distress, sex, and the interaction distress x sex (*b* = 0.350, *p* < 0.001). The direct, unconditional effect of emotional neglect on BMI was smaller, yet remained significant (*p* = 0.005).

We further probed the interaction of sex and distress using the Johnson-Neyman-technique^41^ (see Fig. 3).

**Figure 4:**
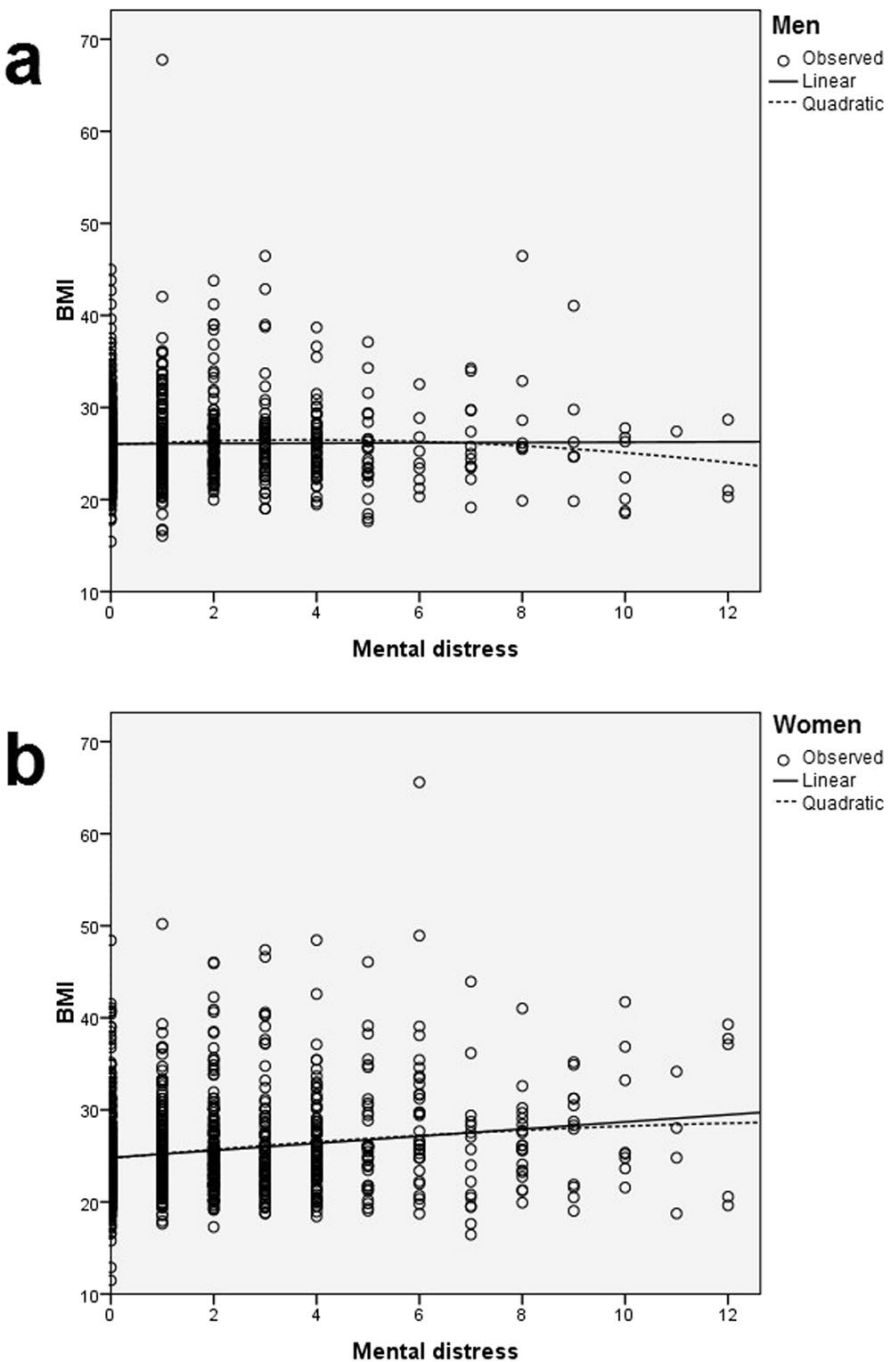
Scatter plots with fitted curves (linear and quadratic) depicting the relationship of mental distress and BMI in men and women. A two-way ANOVA of sex and mental distress on BMI yielded a significant interaction of the fixed effects sex and mental distress (model: *F*(3) = 14.382, *p* < 0.001. mental distress: *F*(1) = 9.768, *p* < 0.001; sex: *F*(1) = 1.918, *p* < 0.001, mental distress by sex: *F*(1) = 11.544, *p* < 0.001). For mean values and SD, see Table 4.

Only in women more distress was related to higher BMI scores (*b* = 0.328, *p* < 0.001). In men, this was not the case (*b* = −0.022, *p* = 0.764). The interaction is visualized in Fig. 4 (Table 4 yields mean values and SD). In summary, this interaction effect in the absence of a significant direct effect of emotional neglect supports the idea of a moderated mediation.

**Table 4:** The effects of mental distress and sex on bodyweight.

				Mental distress			
		Low		High		Total	
BMI		M (SD)	N	M (SD)	N	M (SD)	N
Sex	Men	26.08 (4.09)	1,097	25.96 (5.46)	48	26.10 (4.16)	1,145
	Women	25.23 (4.77)	1,207	27.97 (7.39)	99	25.46 (5.08)	1,306
	Total	25.64 (4.48)	2,304	27.31 (6.86)	147	25.76 (4.68)	2,451

*Note*. Mental distress: PHQ-4 total score, range 0–12. Cut-off according to Löwe, *et al*.^65^: low mental distress: 0–5, high mental distress: 6–12.

## Discussion

In a large, representative community sample, we found associations between maltreatment suffered during childhood and consequences observed in adulthood related to mental distress and bodyweight.

Thus, the present research endeavor replicated previous findings on the association of childhood trauma, in particular of emotional neglect, and mental distress in adulthood^35,42,43^. In the whole sample, we found a relationship between emotional neglect suffered as a child and current mental distress levels across the lifespan. The link of emotional neglect and severe obesity we confirmed in women also corroborates previous research^13,20,22,44^.

Previous research indicated an indirect relationship of childhood trauma and eating disorders in adulthood via emotion dysregulation as previously shown by Racine and Wildes^27^, Moulton, *et al*.^28^ (who had both used the Difficulties in Emotion Regulation Scale). Our model is compatible with these results, placing mental distress (i.e. a manifestation of failed emotional regulation) at the center. We showed that the proposed mediation effect was present in a large, community-based sample with a broad age range, and that it pertained to emotional neglect. Our results also specify that in this sample, the described effect can only be found in women.

Our study differed from previous research which failed to establish a link between childhood emotional neglect and adult weight or eating problems in a number of ways. On the one hand, our participants were older (*M* = 48.4, *SD* = 18.2) than those of the Korean study which investigated adolescents^29^, or the students questioned by Moulton, *et al*.^28^. Also, our sample was drawn from a representative survey, thus it certainly differs from clinical investigations e.g. by^20^ as the overwhelming majority reported no considerable mental distress.

Due to the abovementioned psychometric and socio-historical reasons elaborated by Häuser, *et al*.^45^, we focused on emotional neglect instead of physical neglect. Emotional neglect might be more difficult to verify than physical neglect (as studies investigating physical neglect could also draw from third parties’ records such as Child Protective Services), yet there is broad consensus that emotional neglect measures are relevant as they speak to an atmosphere of emotional invalidation which lay the foundation of psychopathological development e.g.^46,47^. Substantial and long-lasting detrimental effects of emotional neglect have been underscored by the present results.

Importantly, our results highlighted sex differences: The link between mental distress and higher BMI was only found in women. Thus, in women, severe obesity likely is a consequence of disordered eating behavior utilized to cope with adverse subjective emotional states as self-regulation and emotional regulation capacities prove insufficient. This is in line with theories put forth by Cloitre, *et al*.^48^ and Kent and Waller^46^, postulating that an emotionally invalidating environment impedes a child’s capacity to effectively regulate their own emotions, predisposing them to suffer from mental health disturbances later in life. Learned strategies employed to calm or distract oneself when facing distress then include maladaptive ones with negative long-term consequences, such as overeating. Previous research indicated that men and women differ with respect to their preferred coping strategies, e.g. with women favoring emotion-focused strategies previously linked to binge eating^49,50^, and with higher rates of alcohol use disorders in men^51^. Eating has been shown to dampen negative affect and alleviate inner tensions^38,52^. Generally, eating disturbances are part of an internalizing phenotype of psychopathology which is much more common in women e.g.^53^. Perhaps this is why emotional neglect and bodyweight in men were not linked in a statistically significant way. By way of example, a large US study failed to find an association of most types of childhood adversity (including emotional neglect and abuse) with the lifetime prevalence of eating disorders in men^30^.

Additionally, it has been shown that distress is aggravated by a loss of interoceptive awareness and emotion recognition, changes reported in traumatized individuals as well as in eating disorder patients. They entail subdued joy derived from activities which normally contribute to a person’s well-being and positive mood, such as social interactions or hobbies^33,54–56^. The inadequate perception of bodily signals also implicates disturbed appraisal of hunger or satiety, thus influencing food intake^57^.

Furthermore, neuropsychological studies of traumatized individuals have demonstrated diminished capacities to inhibit behavioral actions^58,59^, hence making it difficult to stop eating after having consumed a reasonable amount. Elton, *et al*.^60^ showed that trauma-dependent aberrations of brain circuitry implicated in inhibition differed between men and women. In summary, the results speak to substantial long-term effects of neglect, or “acts of omission”, following the definition offered by Leeb, *et al*.^61^. Therefore, the investigation of childhood adversities should not be limited to “acts of commission” like abuse or violence, but instead include indicators of negative/inadequate caregiver interactions as they also put children at risk for negative sequelae.

A strength of our study is its large, representative population-based sample and its broad age range. Sex-specific analyses are another strong point. Limitations pertain to self-report data with regard to height and weight (used to calculate the BMI), current mental distress, and childhood trauma. Especially the latter could be influenced by memory biases. Previous research attested to the validity^62^ of retrospective, self-reported childhood adversity while an earlier review suggests that there is a bigger risk of self-reports under- than overestimating past childhood maltreatment^63^. Another limitation is that as we investigated mental distress symptoms, the model tests the contribution of risk factors rather than identifying possible resilience factors buffering the impact of childhood adversity e.g. as proposed by current models of risk and resilience^64^. The model explained roughly 7% of the criterion’s variance which means that apart from the factors we included, there should be other major influences on bodyweight across the lifespan. However, the present findings highlight a modifiable risk factor for eating and weight problems which has previously received little attention.

Childhood emotional neglect elevates the risk to suffer from mental distress later in life. However, only in women, this also entailed higher bodyweight. Sex-dependent pathways of emotional neglect on bodyweight via mental distress might be ascribed to different coping strategies and neurocognitive inhibitory capacities. The association between childhood adversity and lower weight in men is less clear. As research on eating psychopathology has often focused on women, less is known about disturbed eating and other factors impacting weight gain and weight loss in men. Our results suggest that for men, these might be different than for women.

With the present study, we confirmed links between different types of childhood abuse/neglect and the lowest and highest BMI categories. Moreover, we found notable sex differences to the effect that for women, childhood emotional neglect was linked to severe obesity, eliciting a possible mechanism behind women’s higher vulnerability towards eating disorders. For men, this connection could not be confirmed. Instead, in men physical neglect was linked to being underweight. Consequently, future research should aim to clarify the pathways of emotional neglect and eating disorders and weight extremes in men and women alike. Both prevention and treatment approaches could benefit from this knowledge.

## Methods

### Participants

From September to November 2016, a representative sample of the German population was surveyed by the demographic consulting company USUMA (based in Berlin, Germany). Participants were chosen via a random route procedure. To be included, individuals had to be 14 years of age or older and to have sufficient understanding of the German language. The final sample used in the present investigation was representative of the German population regarding age, sex, and geographic region. Out of 4,902 designated addresses, 2,510 households participated. Persons in multi-person households were randomly selected using a Kish-Selection-Grid. All participants provided informed consent. In the case of minors, participants gave informed assent with informed consent being provided by their parents/legal guardians. Responses were anonymous. Socio-demographic information was obtained in an interview-format by the research staff and all other information was provided via a questionnaire (handed out with by a sealable envelope). Completed questionnaires were linked to respondent’s demographic data without containing any identifying information. The study was conducted in accordance with the Declaration of Helsinki, and fulfilled the ethical guidelines of the International Code of Marketing and Social Research Practice of the International Chamber of Commerce and of the European Society of Opinion and Marketing Research. The study was approved by the Ethics Committee of the Medical Department of the University of Leipzig. In total, 2,510 individuals took part (1,171 men and 1,339 women).

### Measurements

Childhood trauma was measured using the Childhood Trauma Questionnaire (CTQ). The CTQ comprises five subscales: emotional abuse, emotional neglect, physical abuse, physical neglect, and sexual abuse. Each of the 28 items (e.g. “I had to wear dirty clothes”, assessing physical neglect) is scored on a five-point Likert scale (ranging from 1 = never to 5 = very often). The CTQ has been widely used in community samples as well as in clinical practice and research. Klinitzke, *et al*.^5^ confirmed its 5-factor-structure and attested to its good internal consistencies ranging from Cronbach’s α = 0.62–0.96. Cut-off scores used in this investigation follow norms provided by Häuser, *et al*.^45^ (rating each type of maltreatment “none-minimal”, “minimal-moderate”, “moderate-severe”, or “severe-extreme”). For the present investigation, these categories were transformed into dichotomous variables indicating the presence (i.e. at least moderate-severe scores) of physical and emotional neglect. The 4-item-version of the Patient Health Questionnaire (PHQ-4) was used to measure *mental distress*^65^. As a widely used 4-item screening tool of anxiety and depression, it combines GAD-2 and PHQ-2. It begins with the question: “Over the last 2 weeks, how often have you been bothered by the following problems?”. Response options range from 0 = not at all to 3 = nearly every day, yielding a sum score from 0 to 12. *Bodyweight* categories were calculated according to the WHO’s criteria defining underweight as a BMI (kg/m^2^) < 18.5, and severe obesity as a BMI >= 35^66^.

We calculated equivalised income according to the OECD guideline^67^ by dividing the household income through the square root of people in household. The result was then recoded into the following categories: 1 =< 1250€, 2 = 1250–2500€, 3 => 2500€.

### Statistical analyses

P-values correspond to two-tailed tests. Confidence intervals (CIs) are reported for Odds Ratios (OR) and regression coefficients. Analyses were carried out using SPSS for Windows 24 and the Process Macro by Andrew F. Hayes^68^. We conducted sex-specific binary logistic regressions to test the associations of emotional and physical neglect as assessed by the CTQ and severe obesity/underweight, controlling for age, equivalised income, and the presence of other types of childhood trauma as measured by the CTQ and classified by the proposed cut-offs^45^. We subsequently used a second-stage moderated mediation model (Fig. 1) to ascertain links between emotional neglect, mental distress, and BMI while testing whether this relation was different in men and women. In this model (e.g. described by Edwards and Lambert^69^, the moderator (sex) affects the magnitude of the mediator’s (mental distress) partial association with the outcome (BMI). We entered age, equivalised income, and the sum of the other four CTQ-subscales into the model as covariates. We focused on emotional neglect as a predictor as we aimed to statistically test effects of parental disregard or contempt. For these purposes, researchers have previously cautioned against the interpretation of the physical neglect scale on its own in samples comprising older individuals who lived through/shortly after WWII: At that time, not being provided clean clothing or sufficient amounts of food might have been commonplace and not related to parental neglect^45^.

## Data Availability

The datasets analyzed during the current study are available from the corresponding author on reasonable request.
